# The Maintenance and Adaptability of a Mouse Ascites Tumour (H.A.1) in Roentgen Irradiated Rats

**DOI:** 10.1038/bjc.1958.46

**Published:** 1958-09

**Authors:** Hans-Georg Iversen

## Abstract

**Images:**


### THE MAINTENANCE AND ADAPTABILITY OF A MOUSE ASCITES TUMOUR (H.A.1) IN ROENTGEN IRRADIATED RATS

HANS-GEORG IVERSEN

*From the Department of Pathology, the Finsen Institute and Radium Centre, Copenhagen, Denmark*

Received for publication July 21, 1958

IN animal experiments with different tumours a relationship has been found between the predominant number of chromosomes and the transplantability of the tumour cells (Hauschka and Levan, 1953; Hauschka, 1953; Hauschka and Schultz, 1954; Hauschka, Kvedar, Grinnell and Amos, 1956; Sachs and Gallily, 1956). The host specificity seems to be inversely proportional to the chromosome number of the malignant cells.

Further, in consequence of adaptation of tumours to hosts of different genetic constitution an upward shift of modal chromosome numbers has been demonstrated (Koprowski, Theis and Love, 1956). This might be explained by a selection of cells with high chromosome numbers, as these absorb antibodies to a less degree than cells with lower chromosome numbers (Amos, 1956). But the conditions of an adaptation may also cause genetic changes in the tumour cells (Feldman and Sachs, 1958), and perhaps also stimulate transformations of the latter into cells which are completely compatible with the new surroundings.

The aim of the present study has therefore been to examine whether it was possible to adapt the mouse ascites tumour H.A.1 to a foreign species of animal, and, if so, to search for eventual alterations in the chromosome picture.

The ascites tumour H.A.1, which arose in cortisone treated white mice of the Bagg strain inoculated with human ascitic fluid containing tumour cells (Iversen, 1956, 1958), has up till now (July, 1958) been propagated through more than 130 transfer generations in untreated mice of the strain mentioned. The tumour is highly strain-unspecific and fast-growing, and already after the first two passages in cortisone treated mice, cells with apparent human characteristics disappeared and only cells with murine chromosomes were seen. Simultaneously the ascites tumour could also be carried in untreated animals. It might therefore be that a further chromosome alteration would take place if the tumour could be adapted to a new heterologous host.

In preliminary experiments it appeared that the cells in the ascites tumour were able to proliferate in roentgen irradiated rabbits, guinea-pigs and rats as well as in cortisone treated rats. However, the proliferation was most vigorous iu rabbits and rats. Chiefly for economical reasons, rats were the species chosen for the following experiments.

### MATERIAL AND METHODS

At the beginning of the present investigation, the ascites tumour H.A.1 had been carried through 50 passages in untreated white mice of the Bagg strain, and all the time the tumour cells had shown a triploid stem line number of chromosomes with definite murine morphology (Iversen, 1958).

Inoculations were given intraperitoneally to female white rats of the so-called VS strain bred at the Danish State Serum Institute in Copenhagen ; the animals were two to three months old and weighed about 200 g. Ascitic fluid was always inoculated immediately after tapping of the donor animal, and in quantities of 1·5 ml./rat. In the initial transmission from mice to rats the number of tumour cells in the ascitic fluid was 25 million/ml., and the mitotic index was 3·5 per cent.

The rats were " conditioned ", either by universal roentgen irradiation 24 hours before inoculations (dosage varying between 480 r and 280 r ; 180 kilovolt, 6 milliamp., 0·5 mm. Cu-filter, HVL 1·0 mm. Cu) or by cortisone treatment, beginning 4 days prior to transplantation and continuing with daily doses of 10 mg./rat—also after the transmission.

In the rat passages of the ascites tumour, transplantations were undertaken every 8th day, and for each transfer generation, five treated animals were used. At every fifth passage five untreated rats were also inoculated.

Every fourth day abdominal punctures were done on each animal, and a few drops of the fluid present examined in both haematoxylin-eosin stained samples and aceto-orcein stained squash preparations after the method of Levan and Hauschka (1952) for chromosome studies.

Mice and rats were kept in separate rooms.

### RESULTS

To find a " starting dose " of irradiation and cortisone by which the ascites tumour was able to grow in the rats, one series of animals were irradiated with increasing roentgen doses, and one series of rats with different doses of cortisone (Table I). It appeared that on the 8th day after transmission ascites tumours had developed in the animals pretreated with respectively 440 r and 480 r, but most vigorous growth with the latter dose. A lower dose than 440 r was inadequate to give tumour growth,and in a group of five untreated rats no tumour cell proliferation was detected. In the groups treated with cortisone a progressive growth of

TABLE I.—*Growth of the Mouse Ascites Tumour H.A.1 in Rats " Conditioned " with either Whole Body Roentgen Irradiation in Different Doses on the Day Prior to Transplantation, or Daily Subcutaneous Injections of Cortisone, also in Varying Doses*

| Number of rats | Treatment | Ascites tumour at the 8th day |
|---|---|---|
| 5 | ÷ | ÷ |
| 5 | 280 r | ÷ |
| 5 | 320 r | ÷ |
| 5 | 360 r | ÷ |
| 5 | 400 r | ÷ |
| 5 | 440 r | + (4/5) |
| 5 | 480 r | +++ (5/5) |
| 5 | 4 mg. Cortisone | ÷ |
| 5 | 6 ,, | ÷ |
| 5 | 8 ,, | + (3/5) |
| 5 | 10 ,, | ++ (5/5) |
| 5 | 12 ,, | ++ (5/5) |

(The number of plus signs indicates the rapidity of tumour cell proliferation)

the tumour took place in the animals treated with daily doses of at least 8 mg., but after 10 to 15 days such large hormone doses weakened the animals to such an extent that they often succumbed to different infectious diseases. Apparently, also the tumour cell proliferation was highly compromised by the doses mentioned, as the ascites tumours on the 8th day were only about half the size of the tumours in the irradiated animals. Consequently, as a means of maintaining the ascites tumour in rat passages, cortisone treatment was abandoned, and in the following experiments only roentgen irradiation was used. A "starting dose" of 480 r was chosen.

FIG. 1.—Mortality rate during the 82 rat passages (5 rats/passage) of the ascites tumour H.A.1. The number of dead rats is summed up at every 5th passage. Dot-and-dash line indicates the different irradiation doses.

*The fate of the ascites tumour on lowering the irradiation dose*

After two relatively unsuccessful experiments, where the ascites tumour could only be carried through six and three passages respectively, a permanent rat line of the tumour was established in the third attempt. By transplanting the tumour every 8th day it was carried through 82 rat passages (over more than $1\frac{1}{2}$ years).

In the first ten passages, where an irradiation dose of 480 r was used, the growth of the ascites tumour became gradually more vigorous, indicated by more rapid ascites formation, larger amounts of ascites, and also by increasing mitotic indices. On the 8th day after transmission the mean mitotic index rose from 2·6 per cent in the first passage to 3·1 per cent in the ninth passage (estimated on the basis of 500 cells, i.e. 100 malignant cells from each of the animals).

The irradiation dose thereafter was lowered at every tenth passage, but to secure the tumour against extinction, another group of animals was simultaneously pretreated with the previous irradiation dose. In this way an irradiation dose of 280 r was reached after 50 rat passages (Fig. 1), and in this whole period the tumour grew extremely vigorously. From the 10th to the 50th rat passage the mitotic indices on the 8th day after inoculation were constantly around 3·0 per cent, and at the same time the quantity of ascitic fluid in each animal was estimated to be between 50 and 70 ml. (Fig. 2) with contents of malignant cells of about 20 million/ml. (ranging from 12 to 27 million/ml.).

In the animals which did not die from the tumour formation, the amount of ascitic fluid began to decrease, usually between the 8th and the 12th day after transmission, and tumour regression was always complete within twenty days of the transplantation.

As the regression proceeded the ascites tumour was more and more mixed with lymphocytes and leukocytes, while the number of tumour cells was reduced.

The mortality rate showed a transient fall between the 10th and the 20th passage, presumably because of the reduced irradiation dose, but for the rest of the exprimental period mortality increased steadily. finally reaching nearly 50 per cent (Fig. 1). In most cases the cause of death undoubtedly was due chiefly to the malignant growth, as the autopsies, apart from the ascitic fluid, disclosed solid and semisolid tumour masses surrounding the abdominal organs (Fig. 3), and often infiltrating these (Fig. 4–5).

By lowering the irradiation dose to 240 r the tumour cells proliferated for only two to three days, and none of the rats injected died, while in the animals simultaneously treated with 280 r the proliferation was extremely powerful.

In untreated rats the transplantations never resulted in tumour growth.

At the 70th and the 80th rat passage the irradiation dose was also lowered from 280 r to 240 r, but in no case did the tumour grow.

From the 50th to the 82nd passage the tumour was maintained in rats pretreated with 280 r, which all the time gave a vigorous tumour cell proliferation and a mortality rate of about 50 per cent.

At the 60th passage a parallel experiment was performed by inoculating rats exposed to different irradiation pretreatment with tumour cells from the mouse line of the H.A.1. Again groups of rats (5 in each) received a pretreatment of respectively 280, 320, 360, 400, 440 and 480 r, and on an analogy with the results from the initial experiment, the tumour was able to grow only in the animals which had received 440 and 480 r respectively. By this time, however, the rat line of the ascites tumour had been carried in 60 transfer generations, and was able to proliferate in animals exposed to considerably less irradiation dose, and in these the growth was even faster and more fatal to the animals. Most likely, the ascites tumour through the many rat passages must have obtained a certain degree of adaptation.

As it will appear later, back-transfers to mice were often performed, and the takes and mortality were always 100 per cent.

*Cytological examinations*

Already in the first rat passage a tendency to increased chromosome number in the dominant number of tumour cells was observed. After a few further rat passages this tendency became still more prominent, and at the 5th transfer generation in rats a count of the chromosomes in 50 tumour cells showed a stem-line number between 80 and 90, with a variation from about 50 to 180. This shift from triploidy to tetraploidy (Fig. 6*a*, *b*) corresponded to an increased nuclear size of the tumour cells. The changed modality persisted throughout the many passages of the tumour in rats. Hypertetraploid chromosome numbers were often present, but their frequency was not estimated to be higher than in the original H.A.1 tumour.

As to the chromosome morphology, no gross changes were seen in any of the numerous rat passages of the ascites tumour. The chromosomes maintained the general type of murine chromosomes (Fig. 7). V-shaped chromosomes were not

FIG. 6*a*.—Distribution of chromosome number in 74 cells from the 52nd mouse passage of the ascites tumour H.A.1. A triploid stem line number is seen.

FIG. 6*b*.—Distribution of chromosome number in 81 cells from the 9th rat passage of the ascites tumour H.A.1, demonstrating a tetraploid stem line number.

seen. The author, however, has not felt competent to undertake a more exact analysis of the chromosome structure.

After back-transfers to untreated mice, which were carried out at every 5th rat passage, a reduction of the chromosome number and the average cell size always took place. The chromosome distribution assumed the characteristics of the original H.A.1 tumour. Also, concerning the growth rate and the mortality pattern, the back-transferred tumours were in full accordance with the tumour held in mouse passages only.

In the animals from the 70th, the 71st and the 72nd rat passages, where the malignant growth had regressed, a secondary inoculation was performed one month after the primary and after pretreatment (480 r) on the day before transmission, but the rats were found to be immune.

All rats surviving the tumour inoculation were killed after three months; in no cases could tumour tissue or other pathological changes be demonstrated.

### DISCUSSION AND CONCLUSIONS

It is known that the chromosome morphology in tumour cells can be changed by a variety of cytologic mechanisms (Levan, 1956), and that genetic changes are observed following adaptation of tumours (Feldman and Sachs, 1958).

Changes in the dominant number of chromosomes can be seen after heterologous transplantations of ascites tumours. Perhaps an increased number results most frequently (Koprowski, Theis and Love, 1956), but also reductions in the chromosome number might take place (Ising, 1955). The usual invariable equilibrium between the genetically different cells in transplantation tumours (Levan and Hauschka, 1952; Hauschka and Levan, 1953) might thus be interferred with by alterations in the surroundings.

Also in the present work a distinct increase in the dominant number of chromosomes was seen after transplantation of the ascites tumour H.A.1 from mouse to rat. Simultaneously the tumour to some extent was adapted to the new host, indicated by increased mortality rate among the rats in spite of gradually reduced irradiation pretreatment during the passages. The chromosome structure, however, was apparently not changed. After back-transfers to mice the ascites tumour behaved like the mouse-carried line and at the same time the chromosome number was reduced to the original level. It might therefore be assumed that it was the same type of tumour cells which grew in both the animal species, but that a selection might have occurred among the tumour cells in the rats in favour of cells with a higher degree of polyploidy.

A selection, however, is probably only a step on the way to real adaptation, the completion of which seems to require also some sort of tumour cell transformation. Possibly some assisting factor or agent plays a part in such a transforming process, which might lead to an exchange of cell proteins and finally to chromosomes of altered appearance. Putnoky (1930, 1938) and de Balogh (1940) claimed that alterations in the cell proteins had taken place in the well adapted mouse to rat Ehrlich-Putnoky tumour. That altered cell composition really can occur by the influence of a foreign environment was demonstrated by Langman (1953), who found cat protein in embryonic rabbit tissue grown in a medium containing cat proteins.

Much is in favour of an assisting factor or a transforming agent in the case of the ascites tumour H.A.1 when it developed in cortisone treated mice inoculated with human ascitic fluid containing tumour cells (Iversen, 1956, 1958) The demonstration of human antigens in human cancers carried in several animal passages (Korngold and Lipari, 1955; Korngold, 1956) probably indicates that these tumours had not yet been completely adapted to the heterologous host, which also might be indicated by the facts that the tumours could be grown in "conditioned" hosts only (Toolan, 1953, 1954, 1957), and that they still had human chromosome characteristics (Levan, 1956).

### SUMMARY

The mouse ascites tumour H.A.1 has been successfully propagated through 82 transfer generations in rats pretreated with whole body roentgen irradiation, by inoculating the tumour every 8th day.

The irradiation dose necessary for vigorous tumour cell proliferation was found to be about 480 r. At every 10th rat passage of the tumour the irradiation dose was lowered until a dose of 280 r was reached after 50 passages. A parallel experiment at this time showed that 280 r was insufficient to give tumour growth in rats transplanted with the mouse line of the ascites tumour. Further, the tumour cell proliferation during the rat passages was also more and more vigorous, and the mortality rate increasing—in spite of the gradually decreased irradiation dose. A certain degree of adaptation to the new host was therefore assumed.

Chromosome counts showed that the modality changed from triploidy to tetraploidy after a few rat passages, and the new stem line number persisted through the many subsequent rat passages of the tumour cells.

In the chromosome structure no gross changes were seen. Murine characteristics seemed to be maintained through the passages.

Back-transfers into mice caused a reduction of the chromosome number to the original, triploid, level.

Rats in which the tumour had regressed were found to be immune to further inoculations of the ascites tumour.

In cortisone treated rats the ascites tumour was also able to grow, but the doses necessary were so heavy that both the life of the animals and the tumour itself were compromised.

The results are discussed, with special reference to the adaptation mechanism of heterologous tumour transplantations.

I am indepted to the Senior Pathologist, J. Clemmesen, for helpful advice and to the Director of the Danish State Serum Institute, J. Ørskov, for facilities concerning the supply of laboratory animals.

### REFERENCES

AMOS, D. B.—(1956) *Ann. N.Y. Acad. Sci.*, **63**, 706.
DE BALOGH, E.—(1940) *Amer. J. Cancer*, **39**, 45.
FELDMAN, M. AND SACHS, L.—(1958) *J. nat. Cancer Inst.*, **20**, 513.

---

### EXPLANATION OF PLATES

FIG. 2.—Two rats from the 5th passage showing large amounts of ascites at the 8th day.

FIG. 3.—Dead rat from the 7th passage, ten days after transmission. Ascitic fluid removed. Large solid and semisolid tumour masses are seen, especially in the upper and left part of the abdomen.

FIG. 4.—Infiltrating tumour growth in the outer wall of stomach. From the rat shown in Fig. 3. Haematoxylin-eosin. × 80.

FIG. 5.—Tumour tissue infiltrating the pancreas. From the rat shown in Fig. 3. Haematoxylin-eosin. × 80.

FIG. 7.—Dividing cell from the 67th rat passage of the ascites tumour H.A.1, showing a tetraploid chromosome number and mainly I- or J-shaped chromosomes. Aceto-orcein. × 1150.

2

3

4

5

7

HAUSCHKA, T. S.—(1953) *Trans N.Y. Acad. Sci.*, **2**, 16, 64.
*Idem.*, KVEDAR, B. J., GRINNELL, S. T. AND AMOS, D. B.—(1956) *Ann. N.Y. Acad. Sci.*, **63**, 683.
*Idem* AND LEVAN, A.—(1953) *Exp. Cell Res.*, **4**, 457.
*Idem* AND SCHULTZ, J.—(1954) *Transpl. Bull.*, **1**, 203.
ISING, U.—(1955) *Brit. J. Cancer*, **9**, 592.
IVERSEN, H-G.—(1956) *Ibid.*, **10**, 472.—(1958) *Ibid.*, **12**, 210.
KOPROWSKI, H., THEIS, G. AND LOVE, R.—(1956) *Proc. Roy. Soc.*, B, **146**, 37.
KORNGOLD, L.—(1956) *Cancer Res.*, **16**, 956.
*Idem* AND LIPARI, R.—(1955) *Ibid.*, **15**, 159.
LANGMAN, J.—(1953) *Proc. Kon. Ned. Akad. v. Wetensch.*, C, **56**, 219.
LEVAN, A.—(1956) *Ann. N.Y. Acad. Sci.*, **63**, 774.—(1956) *Cancer*, **9**, 648.
*Idem* AND HAUSCHKA, T. S.—(1952) *Hereditas*, **38**, 251.
PUTNOKY, J.—(1930) *Z. Krebsforsch.*, **32**, 520.—(1938) *Amer. J. Cancer*, **32**, 35.
SACHS, L. AND GALLILY, R.—(1956) *J. nat. Cancer Inst.*, **16**, 803.
TOOLAN, H. W.—(1953) *Cancer Res.*, **13**, 389.—(1954) *Ibid.*, **14**, 660.—1957) *Ibid.*, **17**. 418.


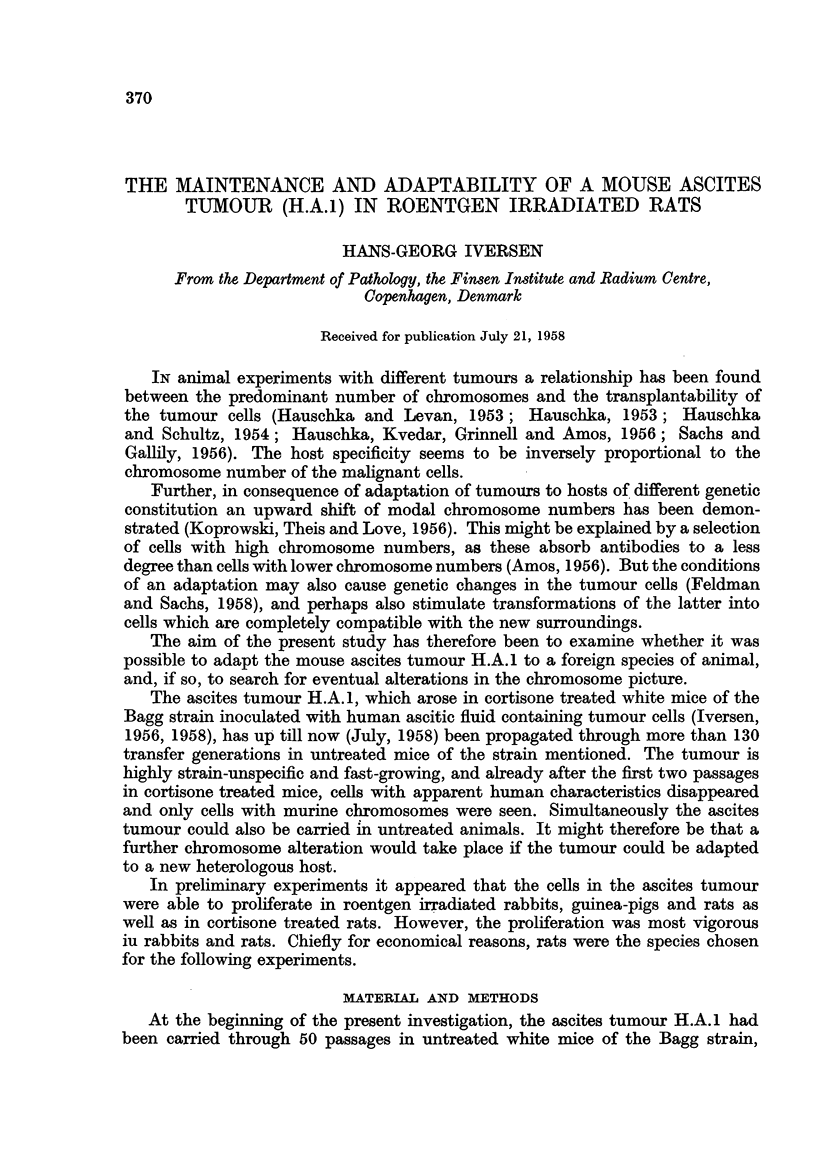

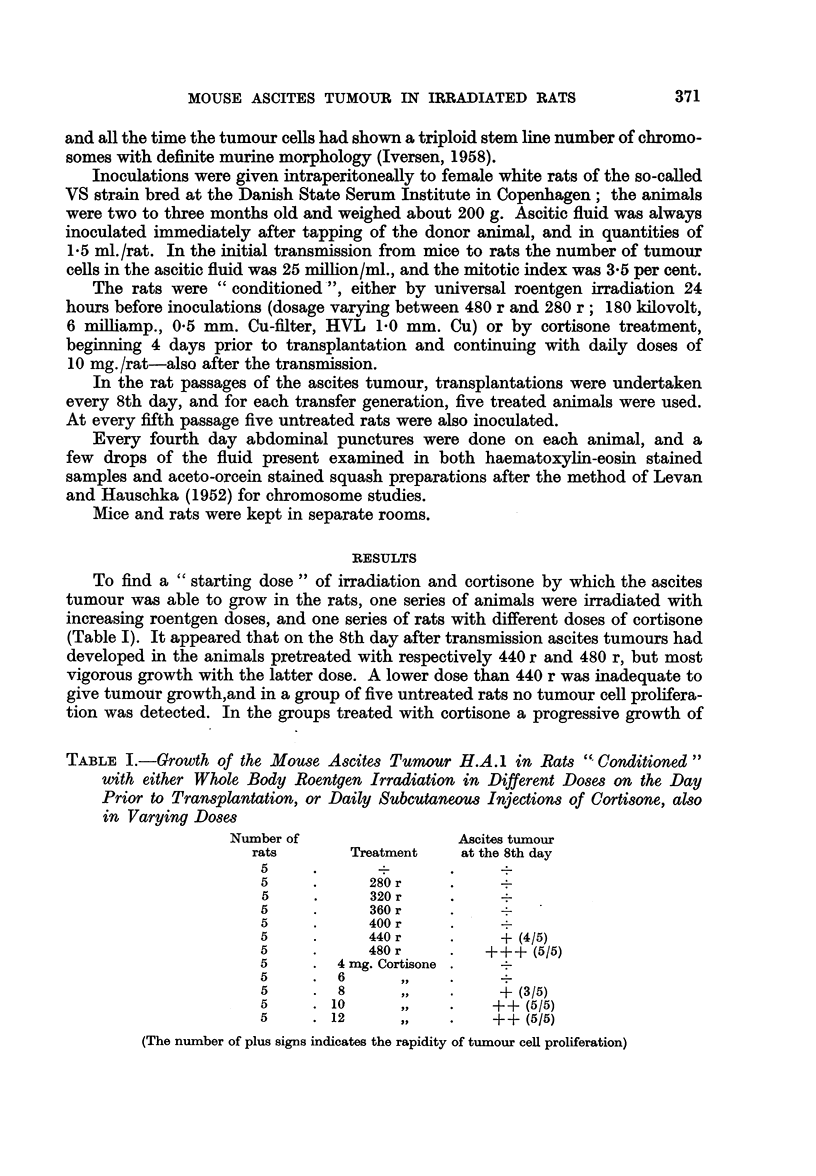

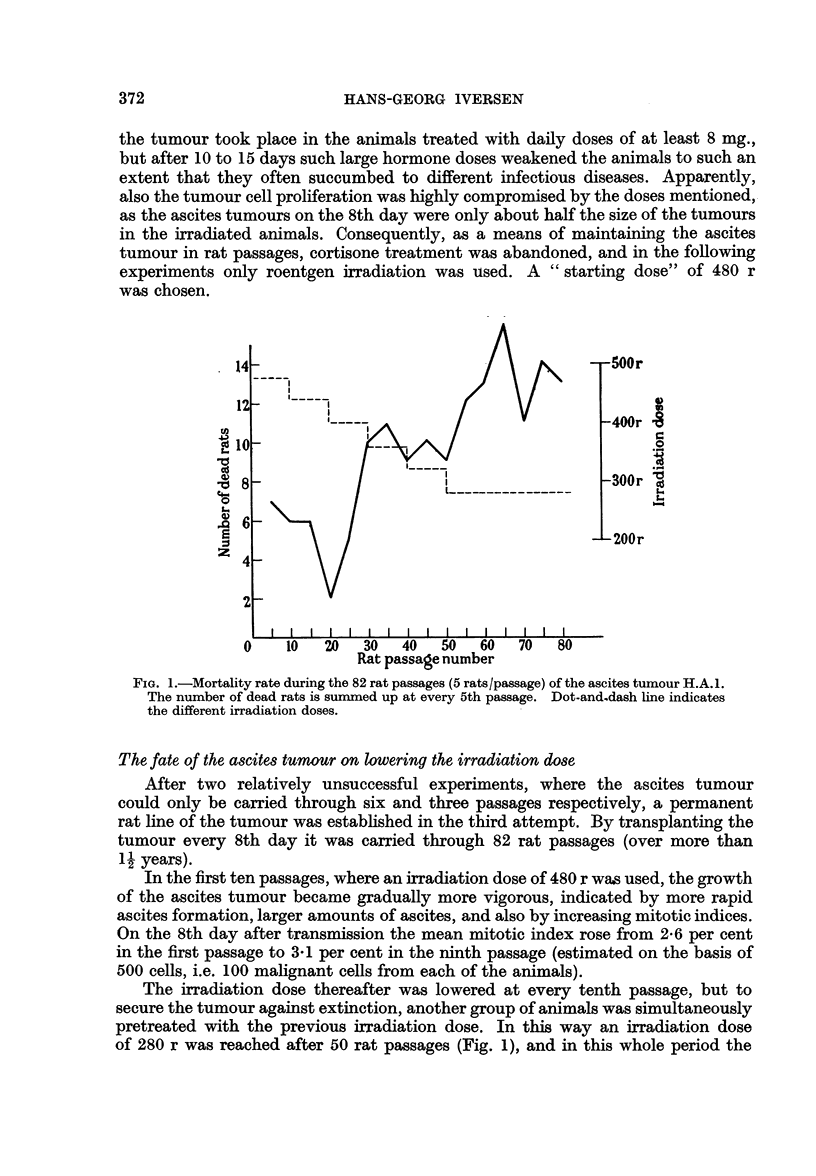

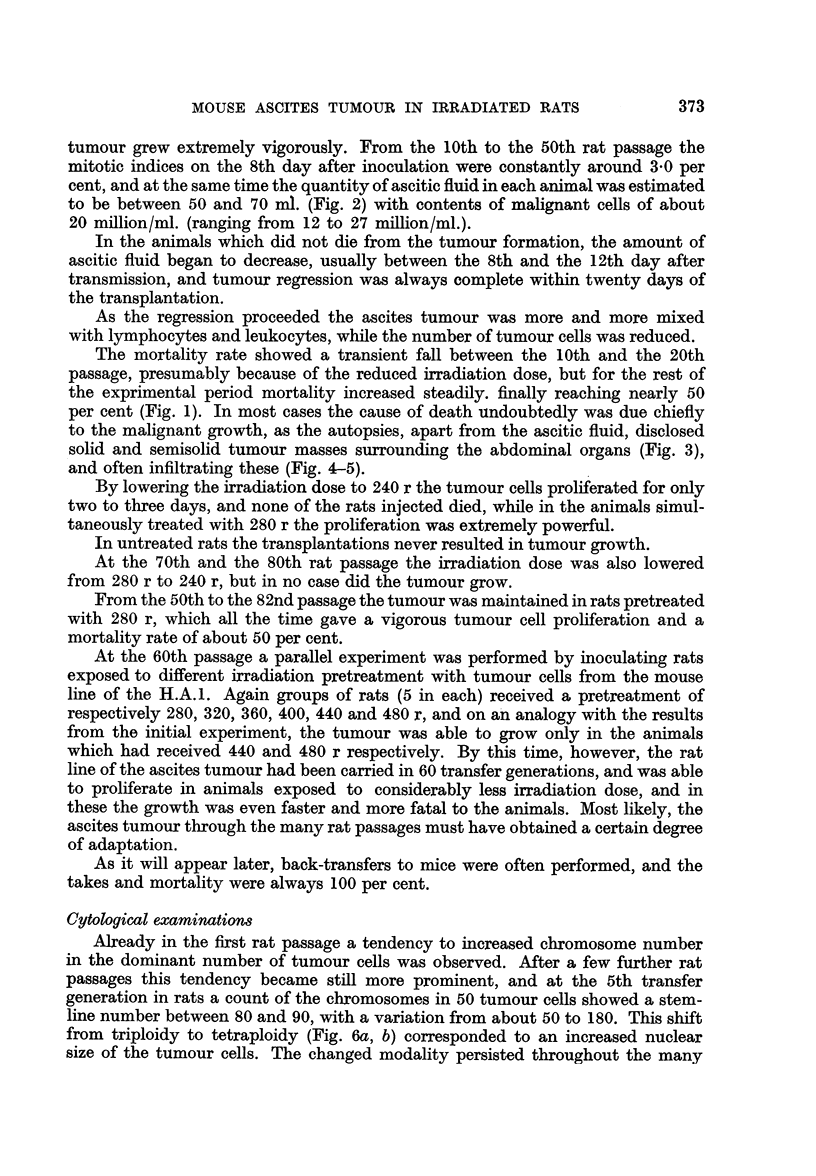

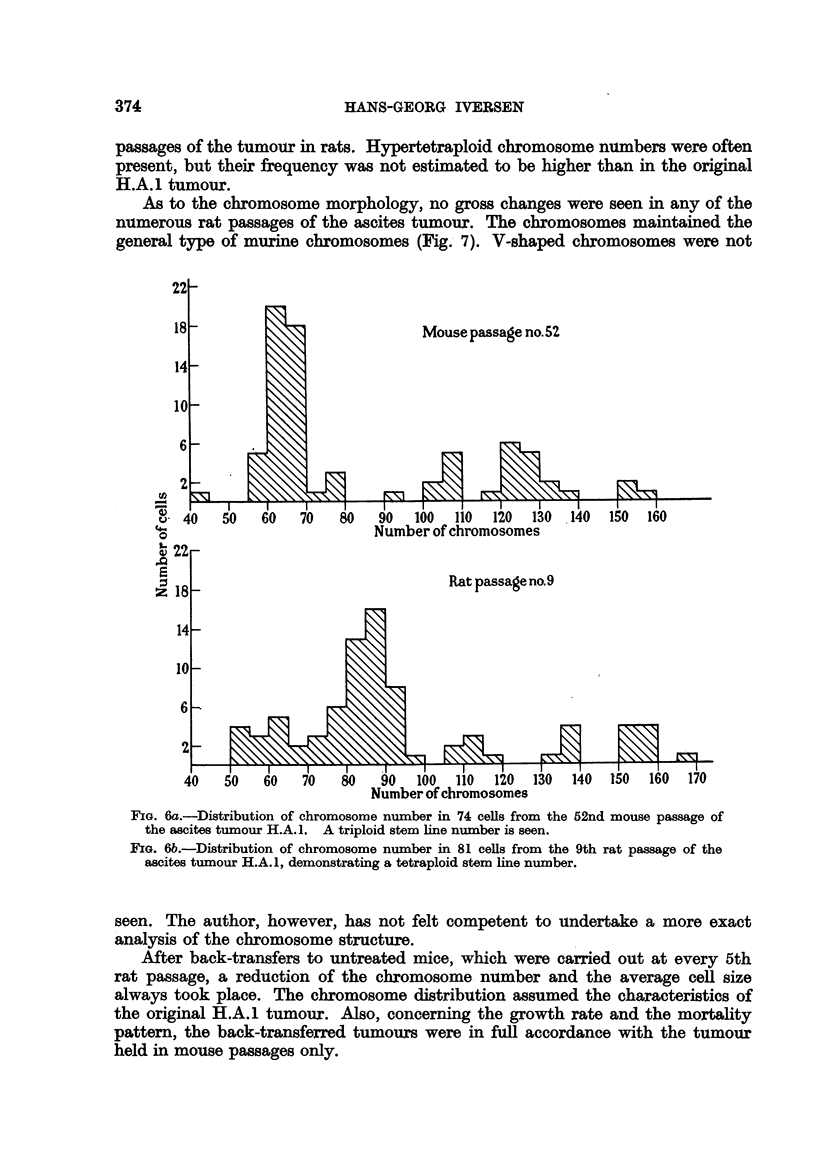

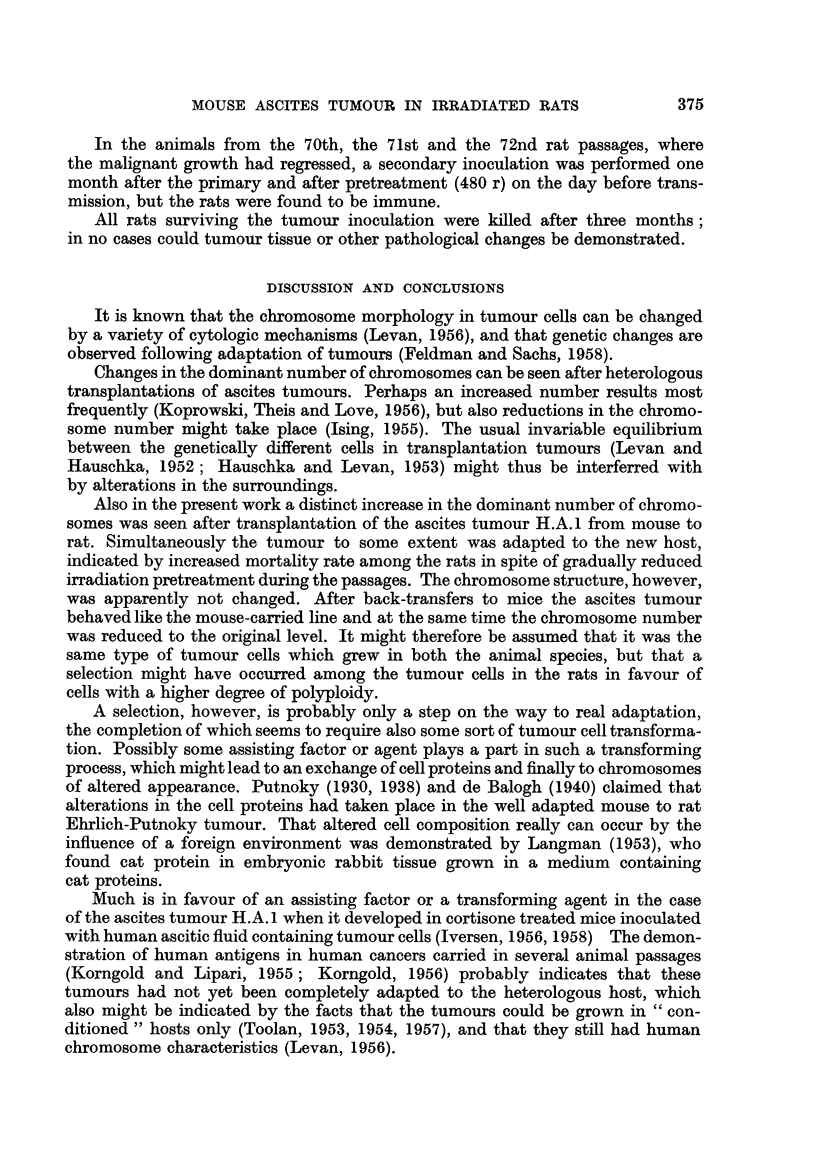

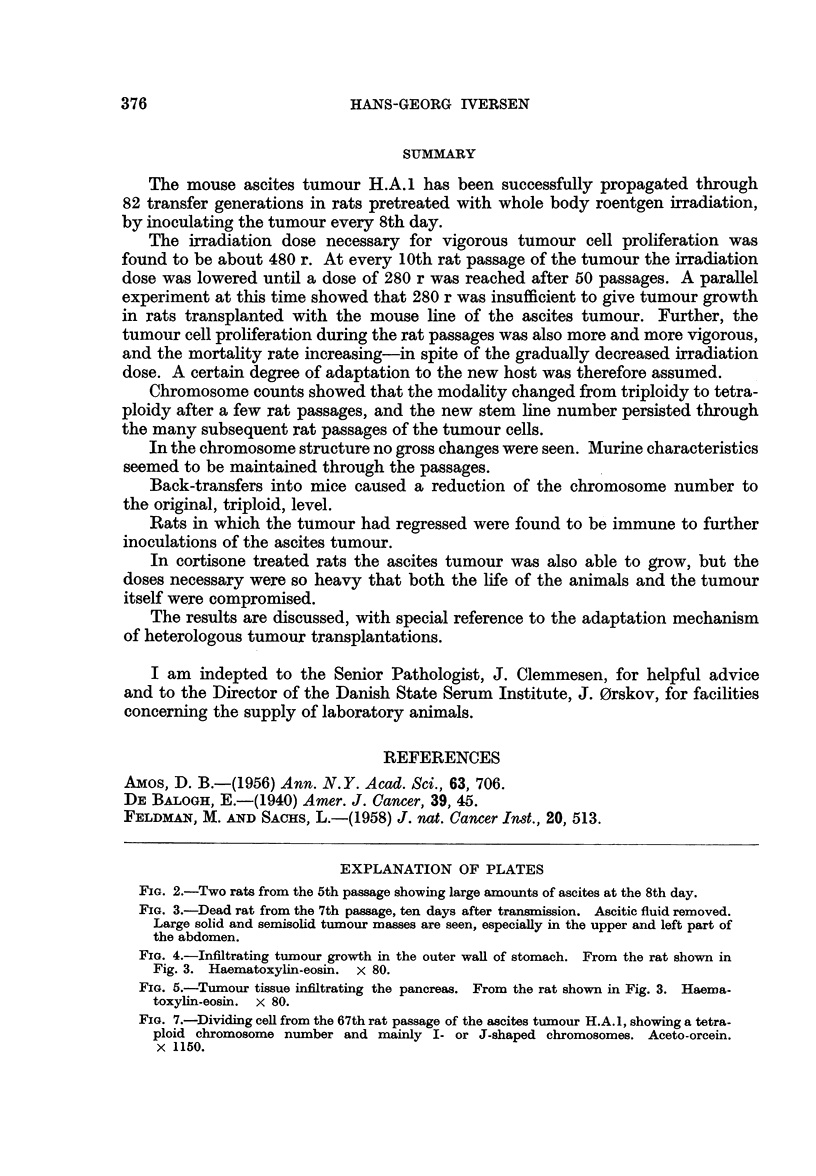

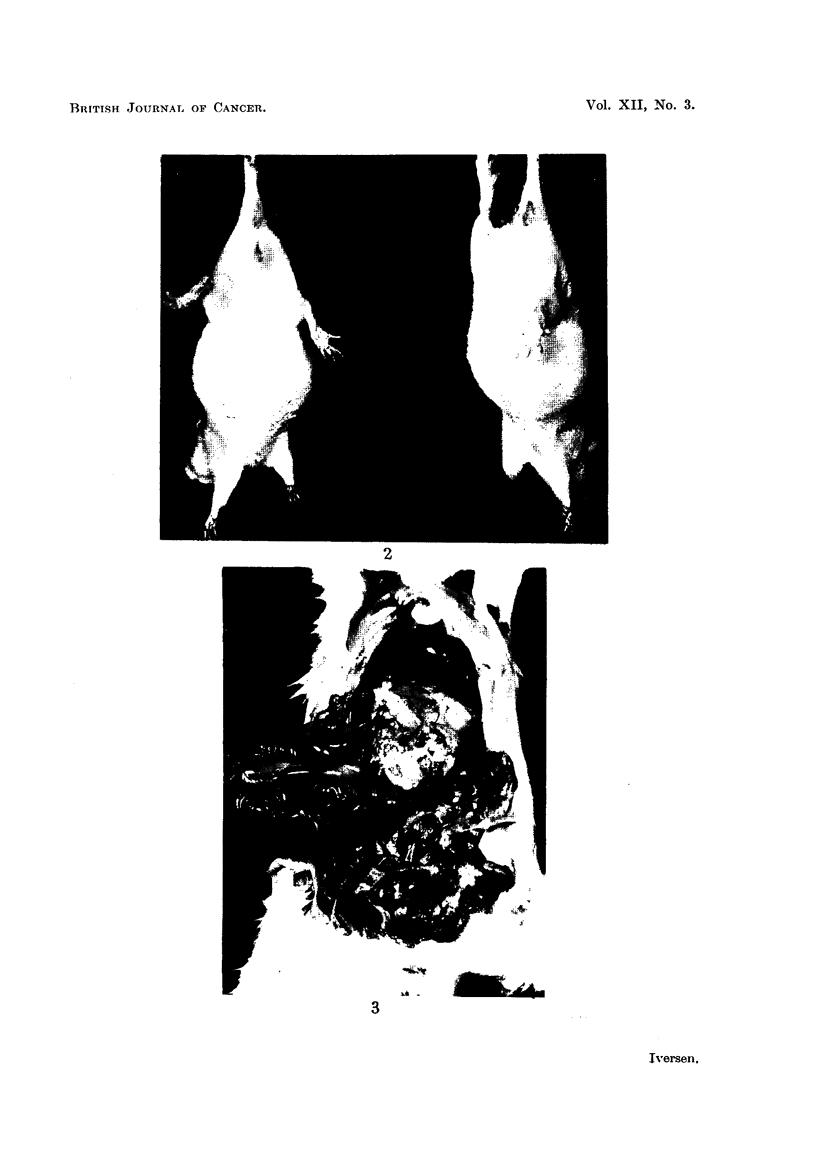

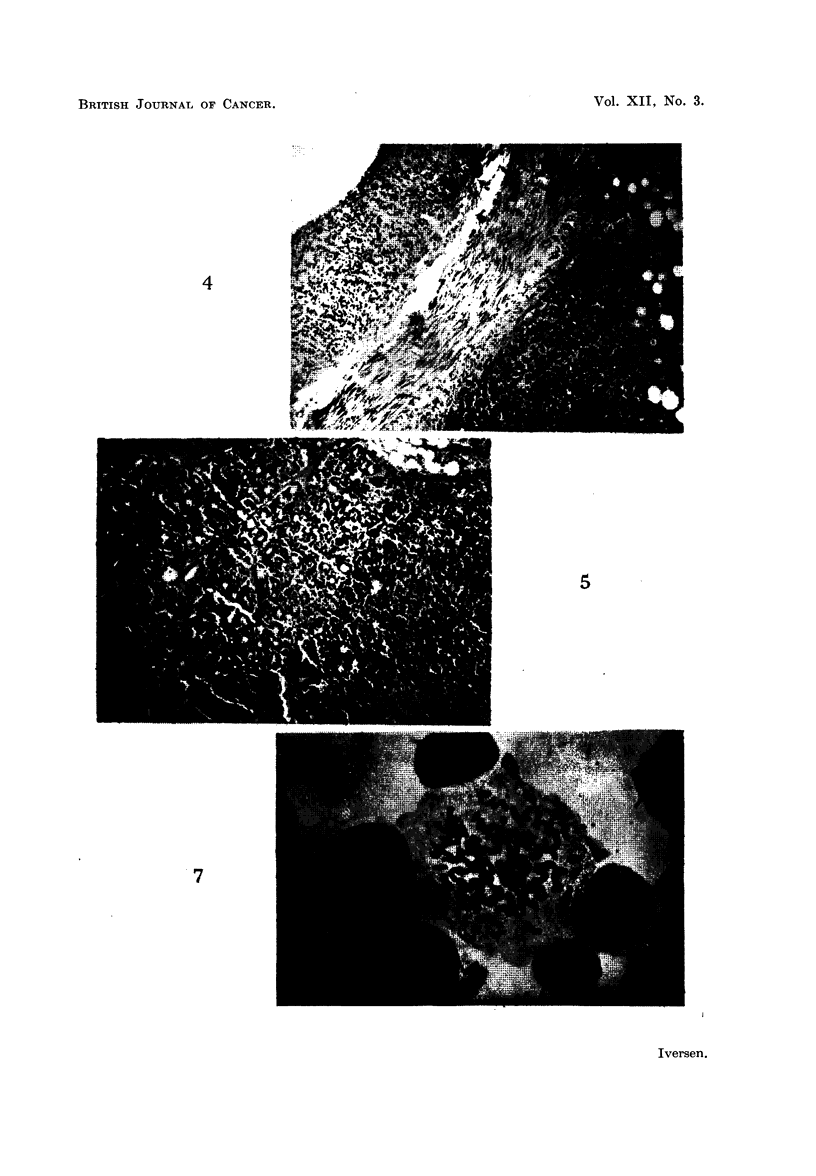

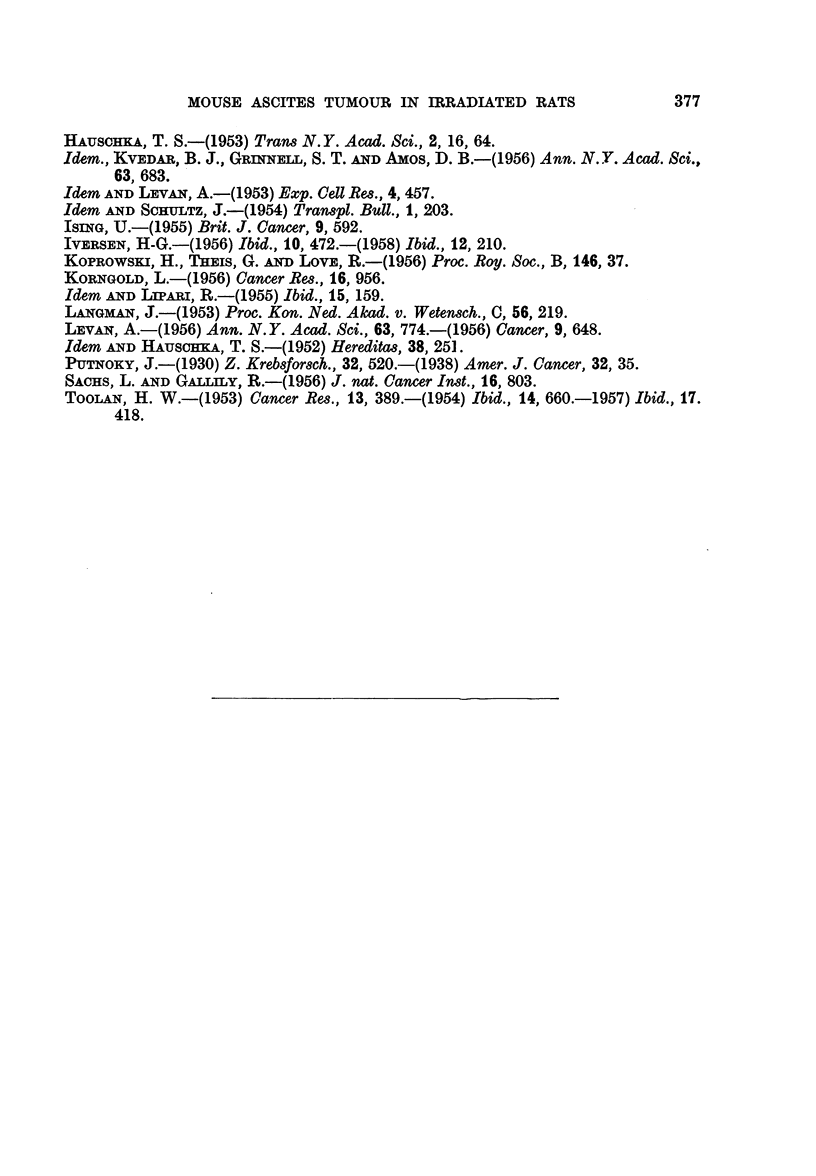


## References

[OCR_00447] AMOS D. B. (1956). Serological differences between comparable diploid and tetraploid lines of three mouse ascites tumors.. Ann N Y Acad Sci.

[OCR_00484] FELDMAN M., SACHS L. (1958). Immunogenetic properties of tumors that have acquired homotransplantability.. J Natl Cancer Inst.

[OCR_00496] HAUSCHKA T. S., KVEDAR B. J., GRINNELL S. T., AMOS D. B. (1956). Immunoselection of polyploids from predominantly diploid cell populations.. Ann N Y Acad Sci.

[OCR_00501] ISING U. (1955). Chromosome studies in Ehrlich mouse ascites cancer after heterologous transplantation through hamsters.. Br J Cancer.

[OCR_00506] KORNGOLD L. (1956). The distribution of human tissue antigens in five human tumors grown in rats or hamsters.. Cancer Res.

[OCR_00511] LEVAN A. (1956). Chromosomes in cancer tissue.. Ann N Y Acad Sci.

[OCR_00515] SACHS L., GALLILY R. (1956). The chromosomes and transplantability of tumors. II. Chromosome duplication and the loss of strain specificity in solid tumors.. J Natl Cancer Inst.

